# Insecticide-treated bed nets and residual indoor spraying reduce malaria in areas with low transmission: a reanalysis of the Maltrials study

**DOI:** 10.1186/s12936-024-04894-2

**Published:** 2024-03-04

**Authors:** Taye Gari, Bernt Lindtjørn

**Affiliations:** 1 School of Public Health, Hawassa University, Hawassa, Ethiopia; 2 Centre for International Health, University of Bergen, Bergen, Norway

**Keywords:** Cluster-randomised controlled trial, Incidence, Indoor residual spraying, Long-lasting insecticidal nets, Malaria, Vector control, Ethiopia

## Abstract

**Background:**

The malaria incidence data from a malaria prevention study from the Rift Valley, Central Ethiopia, were reanalysed. The objective was to investigate whether including an administrative structure within the society, which may have required consideration in the protocol or previous analysis, would provide divergent outcomes on the effect measures of the interventions.

**Methods:**

A cluster-randomized controlled trial lasting 121 weeks with 176 clusters in four groups with 6071 households with 34,548 persons was done: interventions combining indoor residual spraying (IRS) and insecticide-treated nets (ITNs), IRS alone, ITNs alone and routine use. The primary outcome was malaria incidence. A multilevel negative binomial regression model was employed to examine the impact of the kebele (smallest administrative unit) and the proximity of homes to the primary mosquito breeding sites as potential residual confounders (levels). The study also assessed whether these factors influenced the effect measures of the interventions.

**Results:**

The study's initial findings revealed 1183 malaria episodes among 1059 persons, with comparable effects observed across the four intervention groups. In the reanalysis, the results showed that both ITN + IRS (incidence rate ratio [IRR] 0.63, P < 0.001) and ITN alone (IRR 0.78, P = 0.011) were associated with a greater reduction in malaria cases compared to IRS (IRR 0.90; P = 0.28) or the control (reference) group. The combined usage of IRS with ITN yields better outcomes compared to the standalone use of ITN and surpasses the effectiveness of IRS in isolation.

**Conclusion:**

The findings indicate that implementing a combination of IRS and ITN and also ITN alone decrease malaria incidence. Furthermore, there was an observed synergistic impact when ITN and IRS were used in combination. Considering relevant social structures as potential residual confounders is of paramount importance.

*Trial registration:* PACTR201411000882128 (08 September 2014).

## Background

In 2019, a cluster-randomized, controlled trial from the Rift Valley area of Ethiopia was published to determine whether the combined use of long-lasting insecticidal nets (LLINs) and indoor residual spraying (IRS) with propoxur offers greater protection against *Plasmodium falciparum* or *Plasmodium vivax* in all age groups compared to LLINs or IRS alone [1]. The results demonstrated that malaria incidence was comparable among the study groups and that using LLINs and IRS alone or in combination may not eradicate malaria in areas with low malaria incidence [1]. In the same study, an entomological investigation determined that IRS + LLIN were as effective as IRS alone in reducing the human biting rates (HBRs) of *Anopheles arabiensis*, the principal malaria vector in Ethiopia. Moreover, the efficacy of IRS and LLINs in reducing vector density and HBR was greater than that of LLINs alone [2].

At the time of the study, there was a discrepancy between the epidemiological and entomological findings; residual malaria transmission (malaria transmission beyond the reach of standard LLIN and IRS) and insecticide resistance could partially account for some of these differences [1, 2]. The study by Ellie Sherrard-Smith et al*.,* which relies in part on the entomological data from this study, demonstrates that outdoor biting is higher in Ethiopia than in other African countries. They suggest that high outdoor biting combined with a high prevalence of insecticide resistance to LLIN may explain the lower efficacy of household interventions such as IRS and LLINs [3].

This cluster-randomized controlled trial used *gare* (clusters of 30–50 households) as the randomization unit. According to the initial analysis, the intervention groups were well-balanced concerning baseline variables and were analysed according to the study protocol [1, 4]. However, a recent study conducted in the same region with pregnant women recruited from the same study population revealed that some of the variances in outcomes, such as birth weight and linear child growth, could be explained by factors associated with the place of residence, in this case, the kebele (the lowest administrative unit in Ethiopia, and contains a health post staffed by two health extension workers), even if that level was not a part of the selection process [5]. As a result, a reanalysis of the malaria incidence data from the malaria prevention trial was done using multilevel mixed-effect regression models to determine if an administrative structure in the society that needed to be accounted for in the study protocol or first analysis would produce different results.

## Methods

This study represents a reanalysis of previously published and publicly available data, and the study design has previously been described [1, 4, 6]. In brief, a cluster-randomized, controlled trial was conducted in the Rift Valley area of Ethiopia from September 2014 to January 2017 (121 weeks). Forty-four villages or *gares*, each containing, on average, 196 people living in approximately 30–50 households, were randomly allocated to each of four study arms: LLINs + IRS, IRS, LLINs, and control. Each week, 6071 households with 34,548 persons were surveyed by active and passive case detection for malaria.

### Study setting

This study was conducted in the East Shewa Zone of the Oromia Regional State in the Ethiopian district of Adami Tulu. Most of the population lives on subsistence farming or rearing cattle, with a small percentage depending on fishing in Lake Zeway. The lowest level of local government organization in the nation, the kebele, is further subdivided into gares or villages, and together, they make up the woreda (a district; the third level of the administrative divisions of Ethiopia – after zones and the regional states. The woreda is further subdivided into kebeles). The woreda is divided administratively into 48 kebeles, each with a population of 1000 and 5000 people. All 13 kebeles close to Lake Zeway were purposely chosen based on a pilot study [7]. These kebeles were mapped, and 176 gares selected randomly from the 207 gares in the study area were included [1, 4, 7].

In 2016, the district had 104 health extension workers (HEWs) and 43 health posts. According to the National Health Extension Programme (HEP) established in 2007, each district's 48 kebeles shall have at least one health post staffed by two women HEWs reporting to the health facility. HEWs offer crucial health promotion and prevention services through outreach and static programming. Malaria control is one of the services provided in addition to family and child health care and immunizations [8]. The district has 12 public health facilities, one hospital run by a non-governmental organization, and one public hospital.

### Data analysis

The primary endpoint was the incidence of malaria. This paper uses a negative binomial regression model (Stata software; (Stata Corp, College Station, TX, USA) *menbreg* command. In the reanalysis, the effect of the kebele and whether the households within one kilometre from the main mosquito breeding sites, Lake Zeway or the Bulbula River, were included as potential residual confounders. Furthermore, the variable representing the mean distance from each household to the lake might be an alternative metric for the closeness to the breeding site and how agricultural practices are carried out in the surrounding area, considering water pumps for cultivating tomatoes.

The outcome variable, malaria episode, was a count variable that follows a Poisson distribution. However, the data did not satisfy the assumption of the Poisson regression model; the variance is equal to the mean. Therefore, a negative binomial regression model was used to account for the overdispersion in the data. Furthermore, multilevel modelling was employed to measure predictors of malaria episodes to adjust for different levels in the data.

Reassessing the original data showed the same results when not performing a multilevel analysis [1]. Furthermore, randomization was reassessed and showed that the intervention groups were randomly distributed across the gares and kebeles.

The original analysis used generalized estimating equations (GEE) to model clustered data. Several other regression models, such as GEE, Cox, logistic, Poisson, and Bayesian regressions, were used in the reanalysis. Although the effect size varies slightly, the overall results agree: when adjusting for multiple levels, the results are similar to the reanalysis, and when not using such an adjustment, the results match the results in the original publication [1].

## Results

The original results showed that 1183 malaria episodes were identified, of which 55.1% were *P. falciparum,* 25.3% were *P. vivax*, and 19.6% were mixed infections of *P. falciparum* and *P. vivax*. The overall malaria incidence was 16.5 per 1000 person-years of observation time (PYO) and similar in the four arms, with 17.2 per 1000 PYO in the LLIN + IRS arm, 16.1 in LLIN, 17.0 in IRS, and 15.6 in the control arm.

Using a negative binomial regression analysis with malaria as the outcome and adjusting for the administrative structure kebele and distance to the main breeding sites, both ITN + IRS (incidence rate ratio [IRR] 0.63; P < 0.001) and ITN alone (IRR 0.78; P = 0.011) were associated with a greater reduction in malaria cases compared to IRS (IRR 0.90; P = 0.28) or the control group (reference) (Table 1). Furthermore, the reanalysis suggests that the combination of IRS and ITN is significantly better than ITN alone and better than IRS alone.

**Table 1:** Unadjusted and adjusted incidence risk ratios using multilevel negative binomial regression

Variable	Unadjusted				Adjusted for level			
	IRR	P value	95% CI		IRR	P value	95% CI	
*Intervention arms*								
IRS + ITN	1.09	0.352	0.91	1.29	0.63	< 0.001	0.52	0.76
ITN	1.01	0.919	0.84	1.21	0.78	0.011	0.65	0.95
IRS	1.05	0.574	0.88	1.25	0.90	0.280	0.75	1.09
Routine	1.00				1.00			
*Age group*								
Under 5 years	1.30	0.001	1.11	1.52	3.19	0.001	1.11	1.52
6–14 years	1.04	0.631	0.90	1.20	0.52	0.602	0.90	1.20
Above 15 years	1.00				1.00			
*Sex*								
Male	1.01	0.831	0.90	1.15	1.02	0.765	0.90	1.15
Female	1.00				1.00			
*Wealth index*								
Poor	1.18	0.042	1.01	1.38	1.25	0.015	1.04	1.49
Middle	1.11	0.198	0.95	1.29	1.12	0.158	0.96	1.31
Better wealth	1.00				1.00			
*Education head of household*								
Illiterate	1.06	0.657	0.83	1.35	1.05	0.720	0.82	1.34
Read and write	1.50	0.005	1.13	2.00	1.34	0.043	1.01	1.78
Primary education	1.23	0.118	0.95	1.59	1.14	0.314	0.88	1.48
Secondary education	1.00				1.00			
Constant	0.03		0.02	0.03	0.01	0.001	0.02	0.05
lnalpha/	1.75		1.53	1.96	1.11		0.86	1.36
Kebele (var_cons)					0.46		0.13	1.61
Kebele > Dist_Rec					0.35		0.11	1.09

When not adjusting for the kebele structure, the results were similar to those presented in the original analysis, with no effect from the interventions. The large difference in the effect size of the intervention groups when using a multilevel analysis suggests that the levels of kebele and distance to the breeding sites are confounders.

The original analysis used a GEE model. In addition, several other regression models (results not shown) were used, such as Cox regression, logistic regression, Poisson regression, and Bayesian models, adjusting for the kebele and distance to breeding sites variable and without such adjustments. Although the effect size varies slightly, the overall results agree that when adjusting for multiple levels, the results are similar to the reanalysis, and the interventions LLINs + IRS and LLINs significantly reduce malaria incidence. The results are similar to the original publication [1] when such an adjustment was not used.

## Discussion

The large-scale randomized trial shows that interventions using IRS + LLIN and LLIN reduce malaria transmission. There is an additive effect between LLIN and IRS, as it provides the best effect measure and is significantly higher than ITN. Thus, the epidemiological and entomological study findings agree more with each other [1, 2] and a recent comparative trial in Africa [9].

As the effect measures changed significantly when accounting for the level variables kebele and distance to the lake, these variables were most likely confounders, which were, unfortunately, not controlled for in the first publication. Therefore, it may be appropriate to ask why accounting for the kebele structure was not done during the initial analysis. This was considered unnecessary then, as all 13 kebeles adjacent to the lake were selected, and the kebele structure was not used for any randomization purpose [4, 7]. Instead, all 207 gares in these 13 kebeles were mapped and randomized 176 gares. The first publication shows that the distribution of the gares and population characteristics was similar for the four intervention groups (see Table 1 in reference [1]). However, as outlined by Rothmans, the number of randomized groups in each group needs to be large (which was the case in this study), and the analysis should consider the effect of clustering by, for example, performing GEE with the necessary variables or multilevel analysis [10].

Malaria control is one of the services offered by HEWs in addition to family and child health care and immunizations [8]. Even if the Ethiopian Health Extension Programme is found in each kebele and is supposed to have similar functions and coverage, the quality of the work varies from one area to another [11, 12]. Such a variation in the delivery of health services could have influenced how the household members used the trial interventions. Moreover, the variable denoting the distance to the lake may serve as a proxy indication for the proximity to the breeding site and how agricultural practices are conducted in the vicinity, given the use of water pumps for activities such as tomato growing.

A strength of this study is that it was based on a random selection of gares: typical rural communities in Ethiopia. Furthermore, the research encompassed a substantial sample size and many clusters characterized by a high level of statistical power, together with a sufficient duration for follow-up. Even if regression models are used for specific purposes defined by the study designs and how their outcomes are assessed, they measure associations [13, 14]. It is, therefore, encouraging that the analyses using different multilevel regression models are comparable.

Nevertheless, the initial malaria incidence rate seen in the study was lower than anticipated. Initially, the study was grounded on a hypothesized impact size indicating a decrease in malaria incidence ranging from 30 to 50%. The reanalysis aligns with the hypothesized effect of this outcome.

As stated in the first report, the verification of malaria cases was conducted at various healthcare facilities, including health posts, health centres, and hospitals. The probability of the study overlooking a significant number of malaria cases is low. The approach employed for identifying malaria cases combined active and passive case detection methods, which entailed deploying competent personnel and using appropriate rapid diagnostic tests (RDTs) for malaria diagnosis. The first study was included in recent Cochrane and other reviews and received a favourable assessment on outcome measures, bias assessment, and examinations on how LLINs were used [15, 16].

Cluster randomized studies operate at an aggregate rather than on an individual level. Future studies must explore the possibility of hidden level confounders when analysing cluster randomized trials. One such example could be maise cultivation. A maise pollen diet increases malaria mosquito survival, and these mosquitoes are more permissive to *P. vivax* and *P. falciparum* [17]. These findings correspond to earlier studies (also from Ethiopia) showing that the intensity of maise cultivation is associated with increased human risk of malaria [18]. Unfortunately, information on the distribution of maise cultivation in the study area was not collected, but it could be a potential confounder that needs to be accounted for. In addition to the identified or discussed layers, social layers could be invisible to the researchers but need to be mapped.

## Data Availability

The trial data set is available on the following website: https://osf.io/nrbqe/.

## Abbreviations

GEE: Generalized estimating equations
HBR: Human biting rates
HEP: National Health Extension Programme
HEW: Health extension workers
ICMJE: International Committee of Medical Journal Editor
IRB: Institutional Review Board
IRS: Indoor residual spraying
LLINs: Long-lasting insecticidal nets.
PYO: Person-years of observation time
RDT: Rapid diagnostic tests
REK Vest: Regional Committee for Medical and Health Research Ethics, Western Norway
